# Natural Background and Anthropogenic Arsenic Enrichment in Florida Soils, Surface Water, and Groundwater: A Review with a Discussion on Public Health Risk

**DOI:** 10.3390/ijerph15102278

**Published:** 2018-10-17

**Authors:** Thomas M. Missimer, Christopher M. Teaf, William T. Beeson, Robert G. Maliva, John Woolschlager, Douglas J. Covert

**Affiliations:** 1 Emergent Technologies Institute, U. A. Whitaker College of Engineering, Florida Gulf Coast University, 16301 Innovation Lane, Fort Myers, FL 33913, USA; rmaliva@fgcu.edu (R.G.M.); jwoolschlager@fgcu.edu (J.W.); 2 Center for Biomedical & Toxicological Research, Florida State University, Tallahassee, FL 32310, USA; cteaf@fsu.edu; 3 Beeson Consulting, Inc., 12836 Kedleston Circle, Fort Myers, FL 33912, USA; beesonconsulting@outlook.com; 4 WSP USA Inc., 1567 Hayley Lane, Suite 202, Fort Myers, FL 33907, USA; 5 Hazardous Substance & Waste Management Research, 2976 Wellington Circle West, Tallahassee, FL 32309, USA; dcovert@hswmr.com

**Keywords:** arsenic, Florida, soils, geologic units, groundwater, exposure, public health risk

## Abstract

Florida geologic units and soils contain a wide range in concentrations of naturally-occurring arsenic. The average range of bulk rock concentrations is 1 to 13.1 mg/kg with concentrations in accessary minerals being over 1000 mg/kg. Florida soils contain natural arsenic concentrations which can exceed 10 mg/kg in some circumstances, with organic-rich soils often having the highest concentrations. Anthropogenic sources of arsenic have added about 610,000 metric tons of arsenic into the Florida environment since 1970, thereby increasing background concentrations in soils. The anthropogenic sources of arsenic in soils include: pesticides (used in Florida beginning in the 1890’s), fertilizers, chromated copper arsenate (CCA)-treated wood, soil amendments, cattle-dipping vats, chicken litter, sludges from water treatment plants, and others. The default Soil Cleanup Target Level (SCTL) in Florida for arsenic in residential soils is 2.1 mg/kg which is below some naturally-occurring background concentrations in soils and anthropogenic concentrations in agricultural soils. A review of risk considerations shows that adverse health impacts associated with exposure to arsenic is dependent on many factors and that the Florida cleanup levels are very conservative. Exposure to arsenic in soils at concentrations that exceed the Florida default cleanup level set specifically for residential environments does not necessarily pose a meaningful a priori public health risk, given important considerations such as the form of arsenic present, the route(s) of exposure, and the actual circumstances of exposure (e.g., frequency, duration, and magnitude).

## 1. Introduction

Exposure to arsenic in drinking water and soils has become a global and regional concern including Florida over the past two decades because of the real and/or perceived potential impacts on public health [1,2,3,4]. Extremely severe health effects have been observed in regions where naturally-occurring arsenic is found at high concentrations in drinking water, particularly in water wells in India and Bangladesh [5,6]. In the United States, naturally-occurring arsenic concentrations have been measured in groundwater that exceed 12,000 μg/L [7]. A survey of arsenic concentrations in groundwater of the United States found that in 30,000 samples collected, 50% had concentrations <1 μg/L, but 10% had concentrations exceeding 10 μg/L [8]. In response to health concerns about arsenic in drinking water in the United States, the U.S. Environmental Protect Agency (USEPA) reduced the drinking water standard for arsenic from 50 μg/L to 10 μg/L in 2001 which matches the World Health Organization health-based recommendation [9].

The occurrence of arsenic in the soils and groundwater in Florida has received a great deal of attention over the past few decades because of potential human exposure to arsenic as land use changes have occurred in response to population growth [10,11,12]. As land is being converted from a variety of agricultural and rural land uses to the suburban and urban environment, the natural ambient background concentrations of arsenic in soils, as well as areas where anthropogenic influxes have enhanced concentrations above ambient background, have raised public health concerns based on potential human exposure to arsenic in drinking water and soils [3,4,10,13,14]. While a considerable research effort has been conducted on arsenic in Florida to establish background conditions in soils and groundwater, the data are scattered through published and unpublished papers and documents. It is also well-known that a variety of arsenic compounds have been extensively used in Florida as pesticides in agriculture since as early as 1893 with later extensive use on golf courses [11,15,16,17,18,19,20,21,22,23].

It is the goal of our research to compile a comprehensive bibliography on natural background arsenic concentrations in the rocks and soils of Florida (see Section 2, Section 3 and Section 4) and on arsenic enrichment of soil and groundwater caused by anthropogenic activities (see Section 5). Section 6 presents detailed information regarding arsenic in sediment, surface water, and groundwater. The text has been structured to first address the natural concentrations of arsenic in the geologic formations and soils of Florida followed by detailed discussion on the anthropogenic enrichment of the ambient arsenic concentrations. In addition, a preliminary assessment of potential health risks associated with various concentrations of arsenic in soils and water within the urban environment is presented in Section 7. The default Soil Cleanup Target Levels (SCTLs) for arsenic in Florida soils to define contaminated sites currently are set at 2.1 mg/kg in the residential environment and 12 mg/kg in the commercial or industrial environments (Chapter 62-777, Florida Administrative Code). The discussion in Section 8 places these action levels in perspective with respect to background levels, and considers whether they are reasonable, practical and necessary within the realm of public health exposure.

## 2. Overview of Natural Global Occurrence of Arsenic

The natural occurrence of arsenic in the Earth’s crust and in the environment of Florida is common and well-recognized. Taylor and McLennan [24] reported the average bulk concentration of arsenic in the continental crust of the Earth to be 1.5 mg/kg which is likely significantly underestimated based on the analyses of various crustal rock types. Basalt and granite are igneous rocks that constitute a large part of the crust and have average arsenic concentrations of 8.3 and 7.6 mg/kg respectively [25]. Shales and muds have an average concentration of about 10.6 mg/kg [26], and sandstones are believed to have a bulk average concentration of 9.1 mg/kg [27]. The combined limestone and dolomite average concentration has been estimated to be 2.6 mg/kg [28]. Based on the higher values for the majority of crustal rocks compared to the low average value of arsenic reported by Taylor and McLennan [24], Price and Picher [29] suggested that the overall average crustal value should be over 10 mg/kg.

The average concentration of arsenic in seawater has been reported to be 3 μg/L [30]. However, more recent estimates show arsenic concentrations in seawater to differ depending on location. Open seawater arsenic has a range in concentrations from 0.5–3 μg/L with a mean of 1.7 μg/L for the aggregated four valence forms of +5, +3, 0, and −3 [31]. The most common form of arsenic in seawater is arsenate. Minerals in contact with seawater either in bottom sediments or in surface contact with seawater, particularly in a reducing environment, tend to be greatly enriched with arsenic. Arsenic also is enriched in the shells of marine mollusks and crustaceans, which influences the commonly observed phenomenon of arsenic in coastal marine sands and other sediments of Florida and other states [32,33].

There is a tendency for naturally-occurring arsenic to accumulate in organic-rich, anoxic environments which can be marine or terrestrial [34,35]. For example, the large arsenic concentrations in West Bengal occur primarily in peaty sediments with associated high concentrations in groundwater [36]. There is a significant association between the co-presence of organic-sediment, iron, and arsenic [37]. During the microbial oxidation of organic matter and iron, arsenic is released into the interstitial water or into the groundwater system, resulting in a major public health issue [38]. In marine limestones, arsenic is commonly deposited with iron minerals, in particular pyrite, which commonly lines fractures or large pores or occurs as framboids [29].

## 3. Geochemistry of Arsenic

Arsenic occurs in typical groundwater environments in either the reduced arsenite (As^3+^) state or the oxidized arsenate (As^5+^) state. Arsenic ions combine with water to form several main aqueous species. Arsenious acid (H_3_AsO_3_), for example, forms by the combination of an As^3+^ ion with three water molecules:

As^3+^ + 3H_2_O = H_3_AsO_3_ + 3H^+^

**Figure 1:**
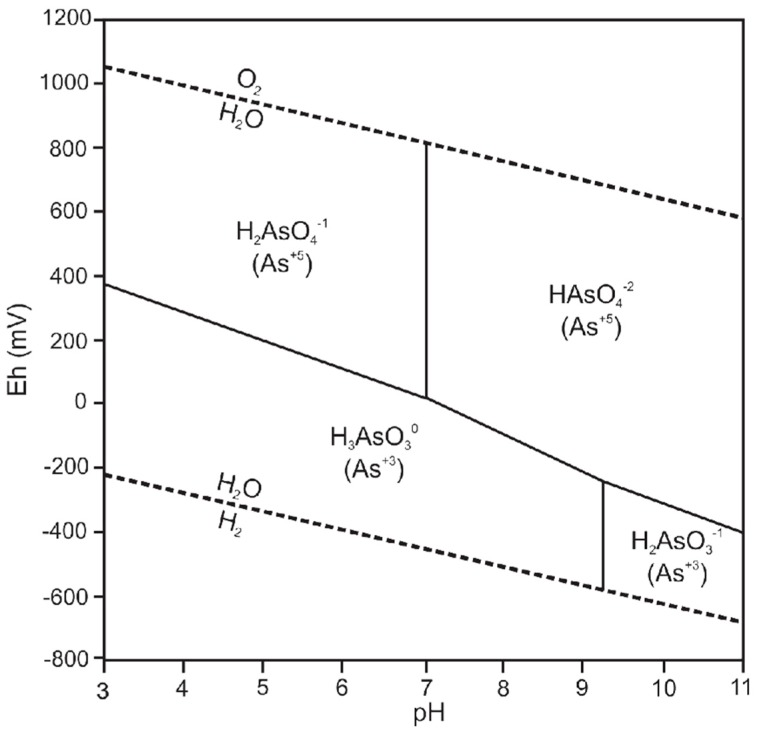
Eh/pH diagram from arsenic in the environment [39].

The thermodynamics of arsenic species and minerals was reviewed by Nordstrom and Archer [39] from whose thermodynamic data an Eh-pH diagram for 25 °C and 1 atm was generated (Figure 1). The H_3_AsO_3_ arsenite species is predominant under reducing conditions and the pH range encountered in normal groundwater. The H_3_AsO_3_ species is not ionized and therefore sorbs less strongly than arsenate species, which results in dissolved arsenite being much more soluble in groundwater than arsenate [39]. Groundwaters with chemically reducing conditions therefore tend to have higher dissolved arsenic concentrations than under oxic conditions, provided that a labile source of arsenic is available in aquifer strata.

Arsenite is thermodynamically unstable in aerobic environments and should oxidize to As^5+^. However, oxidation proceeds very slowly when oxygen is the only oxidant. Other oxidant species, such as iron and manganese oxides, increase the rate of oxidation [40]. Arsenic reactions may also be biologically catalyzed and arsenic species ratios in groundwater may not reflect equilibrium conditions [41,42].

Adsorption is the most significant process controlling arsenic concentrations in most groundwater environments. Adsorption of arsenic is a complex function of the interrelationship between the properties of the solid surface, pH, the concentration of arsenic and competing ions, and arsenic speciation [40]. Oxides of iron, aluminum, and manganese are often the most important sources or sinks of arsenic because of their chemistry, widespread occurrence, and tendency to coat other particles [40]. Absorbed arsenic may be released through competition for absorption sites. Phosphate is particularly effective in promoting the desorption of arsenic. Arsenate and arsenite adsorption are also pH sensitive. Arsenate adsorption is much stronger at lower pH values, with significantly less adsorption occurring above pH 7. Arsenite adsorption, on the contrary, increases with increasing pH, reaching a maximum at between pH 8 and 9 [40].

An important issue in evaluating groundwater arsenic concentration data is the form in which arsenic occurs. Reported arsenic concentration data may consist of dissolved arsenic, arsenic incorporated into insoluble suspended particles, and arsenic sorbed onto suspended particles. Elevated arsenic concentrations due to suspended particles usually are the result of sampling procedures rather than concentrations in the groundwater. For investigations of arsenic in groundwater, gentle, low-flow sampling procedures should be employed during collection of water samples to be analyzed for total and dissolved arsenic. The latter analyses are normally performed by passing the sample through a 0.45 μm filter before addition of a preservative.

Smedley and Kinniburgh [42] reviewed the natural occurrence of arsenic in groundwater and the factors responsible for its mobilization. A key observation is that in most instances of elevated arsenic concentrations in groundwater, the aquifer sediments have near average arsenic concentrations (1–20 mg/kg range), rather than being enriched in arsenic. High arsenic concentrations on a regional scale require both a geochemical trigger that releases arsenic from a solid phase to groundwater, and conditions that allow arsenic to remain in solution in groundwater [42].

Two triggers identified as having led to the release of arsenic on a large scale are the development of high pH (>8.5) under oxidizing conditions in semiarid and arid environments, which causes desorption of adsorbed arsenic from metal oxides or prevents them from being formed, and the development of strongly reducing conditions at near neutral pH conditions leading to the desorption of arsenic from metal oxides, and the reductive dissolution of Fe and Mn oxides leading to the release of sorbed As [42]. As reviewed by Maliva and Missimer [43], arsenic concentrations in the water stored in some aquifer storage and recovery (ASR) systems in Florida are related to redox changes. Dissolved arsenic concentrations in ASR systems appears to be controlled by the introduction of dissolved oxygen during recharge causing the oxidative dissolution of arsenic-bearing iron sulfide minerals. The released arsenic may either stay in solution or be sorbed onto neoformed iron oxy(hydroxides). The sorbed arsenic may be released to solution by the subsequent dissolution or alteration of the iron oxy(hydroxides) by the reestablishment of reducing conditions.

## 4. Naturally Occurring Arsenic Concentrations in Sediments and Soils of Florida

### 4.1. Arsenic Occurrence in Major Geologic Stratigraphic Units

Investigations concerning the arsenic concentration in the major stratigraphic rock units of Florida were initiated because of issues occurring during testing and operation of aquifer storage and recovery projects that use portions of the Floridan Aquifer System to store and retrieve freshwater [29,44,45,46,47,48,49,50,51,52,53,54,55,56,57,58,59,60,61,62]. Treated freshwater injected into and stored in aquifers containing saline water tended to contain enhanced concentrations of arsenic, exceeding drinking water standards upon recovery. Reactions between the oxygen in the injected water, and the induced change in the oxic state of the native water caused the arsenic to be released from the pyrite occurring within the aquifer sediments [56,57].

Large numbers of arsenic analyses were conducted on bulk rock, targeted zones, and individual minerals contained within the Hawthorn Group, the Suwannee Limestone, the Ocala Limestone, and the Avon Park Formation. The compiled results of these analyses are contained in Table 1. When the formations are taken as a whole geologic unit, the average concentrations of arsenic in stratigraphic order are highest in the Hawthorn Group (3–5.6 μg/kg) with the Suwannee Limestone (2–3.5 μg/kg), the Ocala Limestone (1.5–2 μg/kg), and the Avon Park Formation (2.2–3 μg/kg) being lower. The high concentration of arsenic in the Hawthorn Group is likely related to the ubiquitous abundance of nodular phosphate in the unit. Nodular phosphate has an average arsenic concentration of about 7 mg/kg [63]. The geologic units that are composed primarily of limestone show the generally lowest concentrations. There is a major association between the occurrence of pyrite and the occurrence of arsenic with the pyrite grains containing up 11,200 μg/kg of arsenic. The highest concentration of arsenic commonly occurs within large pores or fractures and are associated with the higher abundance of pyrite grains. The pattern of arsenic occurrence follows the global trend of occurrence in anoxic environments associated with iron and perhaps organics (e.g., nodular phosphate).

The arsenic concentrated in the pyrite grains within these predominantly carbonate rocks tends to remain immobile unless the system is exposed to oxygen and/or other oxidizing agents (e.g., nitrate). Injection of oxic water during aquifer storage and recovery operations demonstrates the release of the arsenic from the pyrite [43,45,49,56]. Also, these geologic units constitute a significant part of the Floridan Aquifer System in Florida, where the rocks are located near to the surface and drawdown of water levels in the aquifer could expose the rock to oxygen, creating the potential for natural arsenic release. However, the occurrence of arsenic above drinking water standards in the Floridan Aquifer System has not been found in large areas.

Very limited data are available on near-surface geological units, particularly in southern Florida. Mayorga [64] reported arsenic concentrations of less than 0.2 mg/kg limestone collected from the Miami Limestone sampled at 8 rock mining sites and 22 samples from dragline buckets or rock stockpiles in Miami-Dade County. Solo-Gabriele et al. [12] estimated the average arsenic concentration in the Lake Belt Mining area of Dade County to be 3 mg/kg based on the Arthur et al. [44] estimate in limestones within the Floridan Aquifer System. This estimate is in conflict with the Mayorga [64] analyses. No sampling of bulk rock could be found for the Fort Thompson Formation, Key Largo Limestone, Anastasia Formation, Caloosahatchee Formation, or the Tamiami Formation. Presence of nodular phosphate reworked from the Late Miocene age Bone Valley Formation into the Pliocene Tamiami Formation, Pliocene Cypresshead Formation, the Pleistocene Caloosahatchee Formation, and other surficial deposits resulting from the reworking of phosphate-rich strata make these units of particular interest, because of the relatively high arsenic concentrations found within the Hawthorn Group which is the source unit for the phosphate nodules, and the use of these units as residential water supplies.

### 4.2. Naturally-Occurring Arsenic in Florida Soils

Arsenic concentrations in soils were measured statewide in studies conducted by Ma et al. [65], Ma et al. [66], and Chen et al. [67]. The Florida statewide average concentration of arsenic in soils is estimated to be 1.34 mg/kg based on the work of Chen et al. [67]. However, there are vast areas of Florida where the natural value in soils is higher than the average value and large agricultural or related areas where the values are enhanced based on anthropogenic inputs of arsenic.

**Figure 2:**
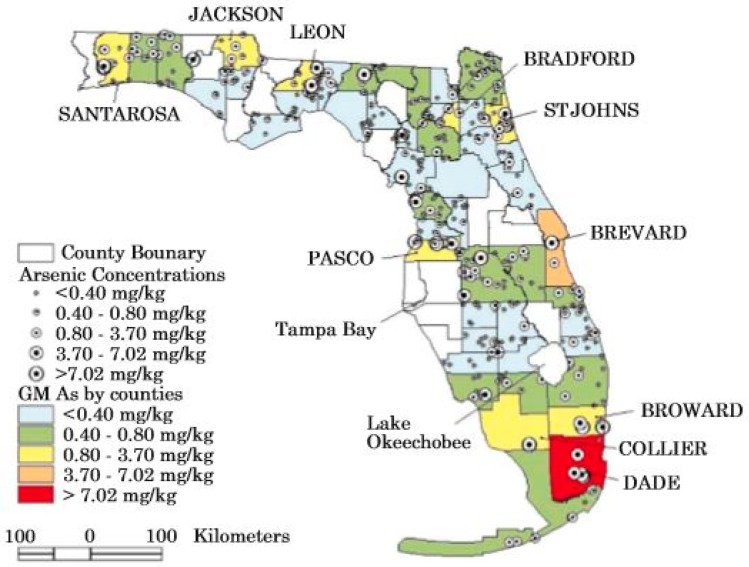
Map showing arsenic concentrations in soils of Florida (from Chen et al. [70]).

A national survey of trace elements in soils was conducted by the U.S. Geological Survey and published in 1984 [68]. A number of soil samples were analyzed in Florida during this survey and provided the first baseline data for arsenic in Florida soils. Several more recent investigations have been conducted on soils of Florida in an attempt to establish the natural background concentrations of arsenic [67,69]. The Chen et al. [67] investigation obtained analyses from 445 soil samples collected from seven soil types located over the entire state of Florida (Table 2; Figure 2). Chen et al. [67] showed that the highest values occurred in histosols (2.06 ± 2.41 mg/kg) and the lowest values in spodosols (0.18 ± 3.23) with the baseline range of values being 0.01 to 50.6 mg/kg. There was a 0.58 correlation coefficient (r-value) to the organic content of the soils.

The relationship between high arsenic content within organic rich soils, commonly occurring in wetlands, was further confirmed in a study of Everglades peats by Duan [71] who found that the mean dry season arsenic concentration in soils was 2.82 ± 1.97 mg/kg and in the wet season was 3.13 ± 2.77 mg/kg. The Duan [71] research is consistent with that of Chen [67] and Ma et al. [66], where he found arsenic concentrations in excess of 50 mg/kg in calcareous endisols. The concentrations in the flocs were higher and in the periphyton were slightly lower (Table 3).

It should be noted that virtually all the Everglades contains soils with concentrations of arsenic greater than the residential SCTL of 2.1 mg/kg. A localized unsaturated soils study in Dade County showed a natural background concentration of 1.2 mg/kg based on 34 analyses [72].

## 5. Anthropogenic Sources of Arsenic in the Florida Environment

**Figure 3:**
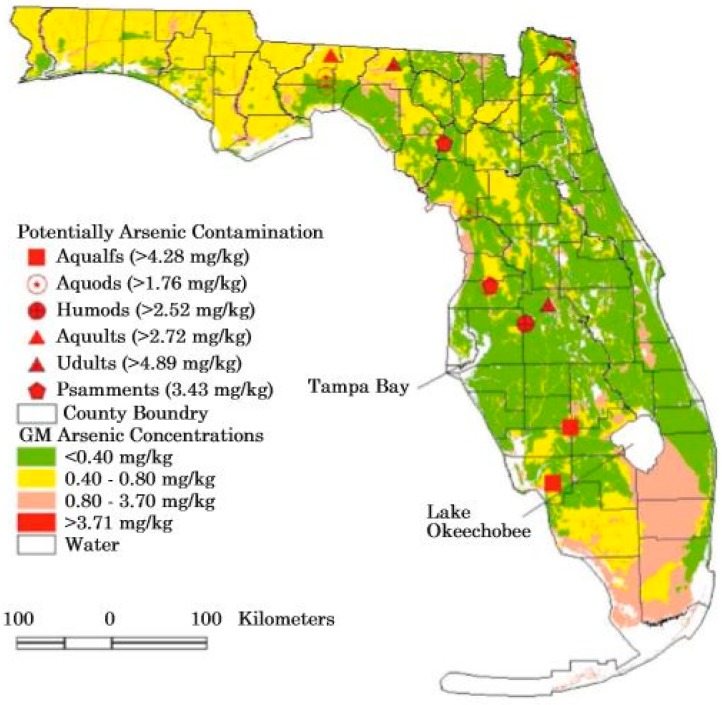
Map showing potential sites contaminated with arsenic in Florida based on soils analyses (from Chen et al. [70]).

The addition of arsenic to the Florida environment is not only a historic issue but is still occurring [12]. Use of arsenic pesticides began in Florida in 1893 or before this time as documented by Lyman Phelps [21]. In 2000, about 2000 metric tons of arsenic entered the Florida environment with 70% added from chromated copper arsenate (CCA)-treated wood, 20% from geologic sources, including the mining of phosphate and limestone, 5% from imported coal, and 4% from the application of the herbicide monosodium methyl arsonate (MSMA) [12]. Since 1970, about 610,000 metric tons of arsenic were used in Florida with 210,000 metric tons for various agricultural applications, 335,000 metric tons for wood-treating, and 65,000 metric tons for a variety of other uses [12]. The amount of arsenic that actually has entered the environment with potential adverse impacts is unknown. Some locations where possible arsenic contamination occurs in soils is given in Figure 3 which is from Chen [70].

Arsenic-containing substances can be classified into two general categories which are wide dissemination associated with legal applications and point sources associated with historic legal uses and recent discharges that may require remedial action. The widely disseminated arsenic sources associated with legal application include arsenical pesticides used on crops, trees, and golf courses, fertilizer use, and soil amendments dispersal (Class AA biosolids, Florida wastewater treatment plant sludge, septic tank solids). Point sources include cattle-dipping vats, wood-treating facilities, litter (chicken) accumulations, and compost.

Industrial and anthropogenic sources are also contributors to environmental occurrences of arsenic in Florida. The largest industrial sources of arsenic in Florida are wood-treating facilities, phosphate-processing facilities, coal-ash, and waste to energy plant ash disposal sites. Wood-treating facilities are also point sources for arsenic, but the disposal of treated wood causes a wider discharge pattern with leaching of arsenic from the wood into stream and rivers, marine waters, and from disposal of the treated wood in landfills and in ash. Phosphate waste sludge, coal-ash, and waste to energy plant ash disposal sites can be considered to be point sources. The last two waste types are commonly placed into landfills which can be unlined (past) or lined (current). Another source of arsenic in the Florida environment is lime sludge from water treatment facilities which can occur as widely-disseminated materials placed on farm fields, or as point locations such as landfills or use as fill material. Arsenic commonly occurs in detention and retention ditches, swales, and ponds used to control urban runoff, natural lake sediments, nearshore marine sediments, and is commonly associated with organic muds.

### 5.1. Agricultural Uses as Crop Pesticides

Arsenical pesticides have been used in Florida since before 1893 to control insects, weed growth, and as a crop desiccant (on cotton) [12,21,23]. Calcium arsenate (CaHAsO_4_) and Paris green (copper acetoarsenite) were used for insect control in orchards, on fruits, tobacco, cotton, and some vegetables and sodium arsenate (NaAsO_2_) was used in cattle dip vats to control ticks, fleas and lice [12]. At a meeting of the Florida State Horticultural Society in 1894, it was reported that Thrip Juice was applied to citrus to kill insects and to sweeten the fruit caused by reduction in acid [23]. Yothers [73] later analyzed Thrip Juice and found that it contained 2.2% arsenic by weight or about 10.12% lead arsenate. Arsenic application was later used in Florida on grapefruit and oranges as a means to eradicate the Mediterranean fruit fly [21]. Some farmers also found that the application of lead arsenate to the fruit hastened the legal maturity of the fruit and allowed it to go to market faster, and also improved the color of the fruit [21,23]. Application of arsenic to citrus trees was found to damage them when the As_2_O_3_ exceeded 2 ppm on new growth on the trees [23]. If the trees were sprayed with bordeaux (a copper sulfide compound), they were not damaged [23]. The Florida legislature banned the use of arsenic on citrus trees, but later allowed its use to control the Mediterranean fruit fly [18].

The older arsenic compounds used as pesticides include arsenic trioxide, lead orthoarsenate, acid lead arsenate (PbHAsO_4_), and lead arsenate which were used extensively on citrus [23]. Some additional products used were Paris Green (copper II acetate triarsenite) and white arsenic. The applications of these compounds were quite concentrated. Singleton [22] reported that a mix of one-half pound of lead arsenate (0.23 kg) with 200 gallons (757 L) of water (227g/757L) yielded good pesticide results. Miller et al. [21] reported accumulations of arsenic in the soils beneath grapefruit tress of 2000 mg/kg in the upper 2.5 to 5 cm of the soil and 6.0 mg/kg at 20 to 25 cm below land surface. They also reported values of 700 and 6 mg/kg of arsenic in soils at similar depths below grapefruit and orange trees respectively. Very high soil concentrations of lead arsenate and arsenic trioxide were also reported at 700 mg/kg. Miller et al. [21] reported minimal leaching of lead arsenate out of the soil. They also found that the concentrations of arsenic trioxide in orange juice in excessively sprayed tress was 0.01 to 0.16 mg/L. It was concluded that this concentration was below a “minimum” medical dose which would make it safe for consumption.

Use of arsenic pesticides in Florida agriculture waned in the 1970’s. However, lead arsenate was used in grapefruit groves into the 1980’s [74]. Some farm areas have had applications of arsenic pesticides for periods of greater than 70 years. Newer organoarsenicals became popular beginning in the 1970’s for use as herbicides [12]. These compounds were used along roadways, railroad right-of-ways, in farms, and golf courses. They included monosodium methylarsonate (MSMA; CH_4_AsNaO_3_), disodium methylarsonate (DSMA; CH_3_AsNa_2_O_3_), cacodylic acid (CH_3_CH_3_AsOOH), and calcium acid methane arsonate (CAMA; CH_4_AsCaO_3_). In 2002, there were 192 products containing active arsenic ingredients registered for sale in Florida (Table 4). MSMA and DSMA were used for weed-control in cotton fields and on golf courses.

### 5.2. Use as Pesticides on Golf Courses

Pesticides containing arsenic have been applied to golf course turf grasses throughout Florida for decades [75]. Chen et al. [76] reported that a survey conducted on 155 golf courses showed that 96% turf grasses were sprayed with MSMA from 2 to 3 times per year with a loading rate of about 224 kg/km^2^. A collaborative investigation conducted by the Dade County Department of Environmental Resource Management (DERM) and the Florida Department of Agriculture and Consumer Services assessed arsenic contamination at five golf courses [77,78]. They found that soil and groundwater contamination was widespread beneath all five sites studied with a maximum concentration of arsenic in the groundwater at 815 μg/L. In addition, the golf course soils contained leachable arsenic that could contribute more arsenic to groundwater [16]. Arsenicals found in the soil can be exposed to oxidation/reduction and methylation/demethylation processes that influence the mobility of arsenic. An experimental investigation conducted by Feng et al. [75] found that the site-specific properties of the soil and transformational chemical processes control the potential for arsenic leaching and movement into groundwater and/or surface-water systems. Soils containing sand grains coated with clay minerals and the presence of organic matter tend to reduce the mobility of arsenic.

The bulk mass of arsenic currently residing in abandoned, old, and new golf courses in Florida is a large value. Solo-Grabriele [12] estimated that the application rate of MSMA ranged between 28 and 897 kg/km^2^ with an average of 190.5 kg/km^2^/application. Based on 1 to 12 treatments per year with an average of 2.5 per year, about 116 metric tons of MSMA was applied. The average concentration of MSMA applied was 1350 mg/L. Ma et al. [79] assessed 11 golf course which had an average soil arsenic concentration of 69.2 mg/kg with a range between 5 and 250 mg/kg in the upper 61 cm of the soil. Ma et al. [79] estimated that 1630 metric tons of arsenic have been deposited on golf courses in Florida. In contrast, the Dade County data analyzed by DERM [78] suggested that the deposition number could be 7160 metric tons of arsenic.

The USEPA adopted a rule to begin phase out of MSMA beginning in 2006 [80] but allowed continued use until an assessment investigation is completed in 2019. The remaining two crops on which MSMA is still used are cotton and golf course turf grass. Regulatory decisions on the use of MSMA are and will continue to be based on its rate of transformation to inorganic arsenic [81]. The agricultural exemption applied to the necessity for remediation based on labelled and permitted use of pesticides may not apply to golf course sites that contain soil concentrations of arsenic above regulatory action levels in the future.

### 5.3. Fertilizers Used in Agriculture and by Home-Owners

The presence of arsenic in fertilizers has been known for decades [82]. Solo-Gabriele et al. [12] identified four fertilizers used in Florida that contain significant concentrations of arsenic. The fertilizers are diammonium phosphate, or DAP (3.8 mg/kg), Ironite (4,777 mg/kg), 13-13-13 (2.8 mg/kg), and 7-3-7 (81 mg/kg). No references were given on how many samples were analyzed for the fertilizer arsenic concentrations studied and overall the database on trace metal composition of fertilizers in Florida is sparse. In Washington State, Woolson et al. [83] found that soils containing elevated arsenic from fertilizer application is related to elevated reactive iron and where reactive iron, and aluminum along with exchangeable calcium are lacking, the arsenic may leach into groundwater. Additional work in Washington State was done by Bowhay [84] to quantify arsenic in fertilizer. A general investigation by Raven and Loeppert [85] found that rock phosphate and phosphate fertilizers contain up to 18.5 and 13.7 mg/kg of arsenic respectively. Dubey and Townsend [86] reported that unacceptable leaching of arsenic into soils in Florida occurs when using the fertilizer Ironite. They reported gross concentrations in three grades of the Ironite fertilizer, including 1-0-0 (2825–3600 mg/kg), 12-10-10 (345–394 mg/kg), and 6-2-1 (0.15–0.23 mg/kg). Research on arsenic in commonly used fertilizers has found that the range in concentration can be 0–85 mg/kg in California [87,88].

### 5.4. Soil Amendments

A variety of organic and inorganic substances have been used in the past to improve soil characteristics to improve crop production. Wastewater treatment plant liquid biosolids have been spread on agricultural fields to increase the organic content of sandy soils and as a means of disposal [89,90]. Septic tank sludge was also applied to farm fields as a means of disposal and to provide soil conditioning for crop improvement [91]. Florida Department of Environmental Protection (FDEP) [92] reported on the chemistry of the biosolids at 694 facilities based on a 1993 inventory of sites where biosolids spreading occurred. Arsenic concentrations in the biosolids had an average concentration of 41 mg/kg with a residual concentration of 20 mg/kg in soils up to a depth of 15 cm [12]. FDEP [92] reported an average concentration of arsenic at 6.1 mg/kg in Florida wastewater treatment facility sludges. With the implementation of Chapter 62-640 in 1998, permits were required for land disposal of biosolids from domestic wastewater treatment plant and septic tank sludges which contained severe restrictions on location of disposal and required monitoring [93]. The ceiling limit on arsenic was set at 75 mg/kg and the maximum average concentration was set at 41 mg/kg. Despite the restrictions, about 88,000 dry metric tons of Class AA biosolids were land applied in 2013 [94]. In addition, about 162,300 dry metric tons of Class AA biosolids were marketed and distributed in Florida in 2013 as soil amendment material [94].

Commercial soil amendments are also used at a smaller scale in domestic gardening. Many of these products, such as Milorganite and others, are produced from dried domestic wastewater treat sludge. Milorganite has a reported arsenic concentration of 4.5 mg/kg [12]. It was found that the Class AA biosolids produced in Florida have an average arsenic concentration of 4.21 mg/kg, the Class B biosolids have an average concentration of 5.68 mg/kg, and the overall concentration of arsenic is 4.80 mg/kg [94]. These concentrations are similar to the value published for Milorganite.

### 5.5. Cattle-Dipping Vats

Historically, “southern cattle fever” was a disease caused by the microbe *Boophilus annulatus* that afflicted cattle in Florida particularly during the early part of the 20th century. Between 1906 and 1963, about 3400 cattle-dipping vats were constructed throughout Florida for the purpose of controlling and eradicating the disease [95,96]. The vats were constructed with concrete with a length of 7.5 to 9 m, a depth of 2.1 m, and a width of about 1 m. A typical vat contained between 5700 and 7600 L of dipping solution that contained 0.14 to 0.22 percent arsenic by weight [97]. The active arsenic compound used in the solution was arsenic trioxide (As_2_O_3_) [12]. Disposal of the spent solution was to direct it into another nearby unlined pit or precipitating the arsenic out of solution by adding iron sulfate and quicklime [98]. The clearing of the used arsenic solution occurred on an annual basis [12]. The sludge was landfilled or spread at land surface. The liquid discharge of arsenic or arsenic-rich sludge resulted in both soil and groundwater contamination. In the later years of use, chlorinated pesticides were added to the dipping solution, resulting in additional contamination with DDT, DDE, or toxaphene at some sites [95]. Use of cattle-dipping vats was discontinued after 1963. Solo-Gabriele et al. [12] estimated that about 1210 metric tons of arsenic were added to the Florida environment by cattle-dipping vats.

A concerted effort was made by the Florida Department of Agriculture and the Florida Department of Environmental Protection in the 1980’s and 1990’s to locate the 3,400 sites, so remedial measures could be taken to remove or confine the arsenic in soil and groundwater. While land-owners were not specifically required to remediate sites, the practical issue of land transfers has necessitated the private remediation of many sites. FDEP [98] published a list of the dipping vats by county in Florida. Woodward-Clyde [99] produced a report to the FDEP that contained a general assessment of the costs to remediate typical cattle-dipping vat contamination sites.

Hydrogeological investigations conducted to characterize and remediate arsenic at cattle-dipping vats have found a wide variety of site conditions with some sites containing primarily soil contamination and others a combination of soil and groundwater contamination. The cattle-dipping vat site found on the Eglin Air Force Base in west Florida contained 2.3 mg/L of total arsenic and 1.1 mg/L of dissolved arsenic in groundwater [100]. The remedial strategy was to excavate and remove soil contaminated with arsenic and allow natural attenuation to remove the dissolved phase. Sarker et al. [101] investigated the effects of soil properties on bioaccessibility of arsenic from sheep and cattle dipping vats.

### 5.6. Chicken Litter

The use of arsenic in commercial chicken feed to stimulate growth has caused the occurrence of some disseminated and point-source impacts [102,103]. The organic compound Roxarsone^®^ (4-hydroxy-3-nitrobenezenarsonic acid) was approved for use in chicken feed beginning in 1944 [97]. The recommended concentration of this compound in poultry feed was 25–59 mg/kg [104]. Momplaiser et al. [105] suggested that virtually all of the Roxarsone^®^ passes through the chickens and ends up in the litter with little or no retention in the chicken product.

Investigations have been conducted on the concentration of arsenic in the chicken litter and in soils that were amended with the litter as fertilizer. Morrison [106] found that chicken litter contained between 15 and 30 mg/kg of arsenic while Lenhart [103] found a higher range between 26 and 51 mg/kg with an average of 38 mg/kg. In a more comprehensive investigation conducted in Georgia, Ashjaei et al. [107] found that chicken litter contained between 14.9 and 26.7 mg/kg of arsenic. In addition, they documented that in a 14-year period of fields using chicken litter for soil amendment, the soils became slightly enriched with arsenic. For the 0–2.5 cm depth range the control concentration was 1.46 mg/kg and the amended field samples showed values of 3.67 and 3.91 mg/kg. For the soil depth of 2.5–7.5 cm, the control value was 1.57 mg/kg with the enriched values being 3.04 and 3.46 mg/kg. Some investigations have suggested that the arsenic in chicken litter has limited mobility in the soils and underlying groundwater based on localized soil conditions with organic matter being particularly important [108]. In Florida, Solo-Gabriele et al. [12] estimated the amount of arsenic produced in chicken manure to range between 2.6 and 3.3 metric tons per year. Most of the chicken litter is believed to be used for land amendment.

### 5.7. Chromated Copper Arsenate (CCA)-Treated Wood and Wood-Treating Sites

A comprehensive investigation of CCA-treatment, use of treated wood, and disposal of treated wood was made by Solo-Gabriele et al. [109], Solo-Gabriele and Townsend [110], Solo-Gabriele et al. [111], and Solo-Gabriele et al. [112]. CCA treated wood is one of the largest sources of anthropogenic arsenic in Florida. The average concentration of arsenic in CCA-treated wood is 1,200 mg/kg and the wood ash may contain an average of 33,000 mg/kg of arsenic [12]. In 2000, there was about 31.2 million cubic feet of CCA-treated wood sold in Florida with a corresponding inflow of about 1,400 metric tons of arsenic [113]. Solo-Gabriele et al. [109] suggested that 60% of the total was treated in Florida and 40% outside of Florida.

The amount of CCA solution added to the wood ranges from 4 kg/m^3^ to 44 kg/m^3^ [12]. They determined that the solution contains about 22% by weight of arsenic which means that the wood produce contains a concentration of between 1700 and 17,000 mg/kg of arsenic. The input of arsenic from CCA-treated wood in 2000 was about 1400 metric tons. A phase-out for residential CCA-treated wood occurred in 2003, but the use was continued for marine and farm applications along with poles, piles, round posts and plywood. Townsend et al. [113] estimated that the total amount of arsenic in CCA-wood “in service” was about 24,300 metric tons.

Therefore, arsenic entering the environment can be disseminated in the form of leaching to surface-water or groundwater or released as point sources from burn piles or used wood or ash placed in unlined construction and demolition (C & D) debris landfills. In addition, old CCA-wood treating sites tend to be significant soil and groundwater contamination sites. CCA-treated wood that is sent to mulching facilities will also add to the disseminated arsenic load. The C & D landfill disposal method was suggested as the largest disposal method used [110,111,112,113,114]. Today, there are specific regulations applied to the placement of CCA-treated wood into landfills.

### 5.8. Lime-Softening Sludge from Lime-Softening Drinking Water Treatment Facilities and Other Water Treatment Sludges

The lime-softening water treatment process is common in Florida because of the use of groundwater from carbonate aquifer systems. This treatment process has been used for potable water production for nearly a century [115]. Based on a study of three utilities, Chen et al. [116] reported that lime-softening sludge contains a dry weight arsenic concentration ranging from 2.4 to 24.6 mg/kg with an associated TCLP arsenic yield of 0.0009 to 0.028 mg/L. Chwirka [117] reported a value of arsenic in softening residuals of 165 mg/kg. Some data on the arsenic content of lime sludge and the arsenic leachability of the arsenic were compiled by Chen et al. [67] (Table 5).

A study of lime sludge chemistry in 19 utilities in Florida was conducted by Townsend et al. [118]. They analyzed the total arsenic concentration and leachability of arsenic in 20 samples which yield an average total concentration of 1.15 mg/kg with a range of 0.18 to 4.94 mg/kg. The leachability of the arsenic was low with 19 samples showing <2.5 μg/L and one sample yielding a concentration of 2.82 μg/L.

In a recent study of a lime softening sludge disposal site in Fort Myers, Florida, the dry weight concentrations of arsenic in 22 samples of the lime softening sludge were measured. Concentrations varied from 1.84 to 21.9 mg/kg with an average of 10.5 mg/kg [119]. EPA Method 1312 Synthetic Precipitation Leaching Procedure (SPLP) tests were performed on 10 of the samples with concentrations that represented the entire range of total arsenic concentrations in the sludge. Arsenic was only detected in one of the 10 SPLP extracts at an estimated concentration of 5.97 μg/L. The concentration of total arsenic in this sample was 10.8 mg/kg [119].

Few investigations have been conducted on the disposal of lime-softening sludge. Nguyen et al. [120] evaluated three methods for disposal of solid forms of the sludge, including landfill, biological treatment by mixing with cow dung, and solidification/stabilization using cement and subsequent land disposal. In Florida, lime-sludge disposal methods have included land-filling of borrow pits (e.g., City of Fort Myers), placement into rock mine pits mixed with residual rock flower from the crushing process, placement in non-hazardous landfills, land application, and use as a soil amendment to reduce pH in farm fields. Sarkar et al. [121] evaluated the immobilization capacity of two Florida soils for removal of arsenic. They studied the use of water treatment residuals to amend soils to increase their adsorption of arsenic. The residuals were primarily Fe/Al (hydr)oxides and not lime-sludges. However, the research was relevant in that the soils containing naturally high concentrations of iron and Al (hydr)oxides will tend to adsorb arsenic at higher rates compared to sandy soils containing these substances in low concentrations. Therefore, lime-sludges can be used as a soil amendment without risk for mobilization in groundwater depending on the soil type. The result would be a higher than natural background of arsenic within the dry weight of some soils. This would be similar to the use of phosphate-based fertilizers, application of biosolids, or soil amendment with manure.

Fe/Al (hydr)oxide and alum sludges also are generated at some existing water treatment plant facilities in Florida. Although concentrations of arsenic in these sludges are generally higher than in lime softening sludges, they are not generated at as many utilities and their disposal is more closely scrutinized.

### 5.9. Concentrated Metals in Urban Storm-Water Management Facilities and Street Sweepings

It has been demonstrated that background arsenic concentrations in urban areas are based on the intensity of development which causes greater migration of non-point source anthropogenic arsenic into soils [122,123,124]. Arsenic concentrations in soils in the Gainesville, Florida area had a range between 0.21 and 660 mg/kg with a geometric mean of 0.40 mg/kg while a more intensely developed area, Miami, had a range of concentrations from 0.32 to 110 mg/kg with a geometric mean of 2.81 mg/kg [125,126,127].

Arsenic in urban areas is commonly concentrated in the Best Management Practices infrastructure to treat urban runoff. Fernandez and Hutchinson [128] investigated the chemistry of bottom sediments in three stormwater detention ponds in Pinellas County, Florida. They found arsenic concentrations ranging from <1 to 3 mg/kg, <1 to 6 mg/kg, and 2 to 15 mg/kg with corresponding average values of 1, 2, and 3 μg/L at the three sites. Arsenic concentrations in the corresponding pond water were <1, 1, and <1 mg/L. Groundwater in the vicinity of the three locations showed arsenic concentration well below the 10 μg/L MCL. An investigation of arsenic accumulation in the sediments of stormwater management systems was conducted by Liebens [129] in northwest Florida. In stormwater pond sediments he found arsenic concentrations ranging from 1.78 to 7.47 mg/kg with a mean of 3.82 mg/kg. Roadside swales had measured sediment arsenic concentrations ranging from 1.58 to 17.68 mg/kg with a mean value of 5.59 mg/kg. The arsenic concentration in street sweepings range from below detection limits to 13.30 mg/kg. Solo-Gabriele et al. [12] reported an average arsenic concentration in stormwater pond sediments of 1.4 mg/kg. However, this value appears to be quite low for commercial land uses which tend to be above 4 mg/kg based on the investigations reviewed [129].

## 6. Arsenic Concentrations in Sediments, Surface-Water and Groundwater of Florida

**Figure 4:**
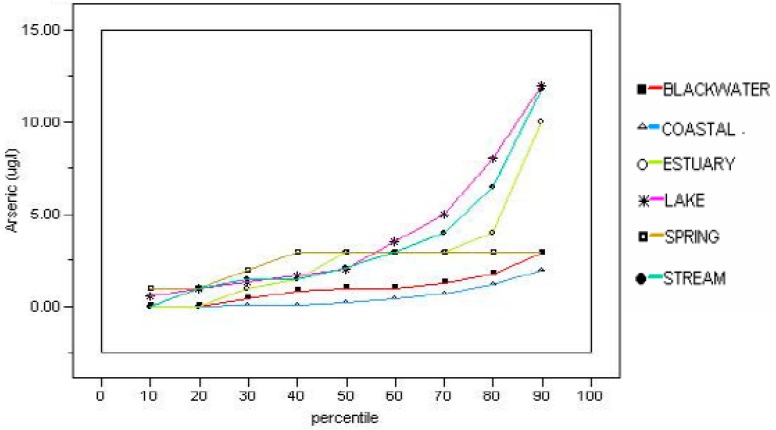
Cumulative frequency curves for arsenic concentrations in various surface-water bodies in Florida (from Hand [135]).

The presence and magnitude of arsenic concentrations in various environmental media are greatly influenced by local conditions, both those of natural origin and those related to human activities. In addition to potential exposure to soil that contains arsenic, exposure can occur from contact with surface water, groundwater or sediment, and the potential hazards will vary with the frequency, duration and magnitude of exposure. In general, human exposure to arsenic via surface water, groundwater or sediment in Florida poses less risk than exposure to contaminated soil. This is due to the treatment of drinking water prior to consumption and the relatively small exposure durations to potentially contaminated surface water and sediment. Therefore, the occurrence of arsenic in these three media are briefly evaluated. Detailed coverage of those issues is beyond the scope of this evaluation, but the reader is encouraged to consult appropriate ancillary references for guidelines regarding how such evaluations are conducted. Following are important, but more general, discussions of the presence of arsenic in surface water, groundwater and sediment follow. Surface-water monitoring of arsenic in Florida has been performed in many areas by the water management districts, the Florida Department of Environmental Protection, the U.S. Geological Survey, and several local governments. Access to these data can be accomplished by searching online at the Florida Department of Environmental Protection STORET site and the websites of the U.S. Geological Survey and the Florida water management districts. Some general compilations have been made with regard to average concentrations of arsenic in surface water bodies as part of the investigations conducted by the Florida Department of Environmental Protection to establish the Total Maximum Daily Loads surface-water regulations. The average concentration of arsenic in Florida Rivers was reported by Solo-Gabriele et al. [12] to be 1.35 μg/L based on data compiled by Coffin and Fletcher [130,131,132,133] for various regions in Florida. An average lake value for arsenic was reported as 3.6 μg/L by Eisler [134]. Hand [135] made a comprehensive study of arsenic concentration in the lakes, streams and estuaries of Florida with an assessment of the frequency of the concentrations (Figure 4). He found that the only concentrations of arsenic above the 10 μg/L MCL at the 90% percentile in surface water in Florida occurred in lakes and streams.

In Florida groundwater, ambient arsenic concentrations are generally low, well below the drinking water MCL. Focazio et al. [136] compiled results of arsenic analyses on 475 groundwater samples in Florida of which 184 samples had concentrations below the detection limit (0.5 μg/L). The maximum concentration detected was 110 μg/L and only 17 samples had concentrations above 10 μg/L. The median concentration of arsenic in groundwater in Florida is about 1 μg/L [12]. Higher concentrations can be found down-gradient from sites contaminated with arsenic from MSMA (e.g., golf courses) [130] and other contamination sites occurring near cattle dipping vats, wood-treating sites, and abandoned agricultural pesticide mixing sites.

Arsenic from soils, surface-water runoff, and groundwater has found its way into lake sediments in Florida. Whitemore et al. [137] found that the concentrations of arsenic in the sediments of Lake Jackson in central Florida averaged 212 times the natural background in the sediments deposited before 1912. Within Pb-210 dated cores they found that peak concentrations of arsenic occurred in sediments deposited in the 1980’s. Arsenic concentrations in the pore waters were 16-43 times the USEPA MCL for arsenic of 10 μg/L. Runoff collected in drainage canals entering the lake was also enriched in arsenic. Arsenic in some water wells located between a golf course and the lake showed arsenic concentrations from below detection limits to 10–11 times the MCL. Since there was no aquatic weed control in the lake that used arsenic compounds, they concluded that the source of the arsenic in the sediments was the use of arsenical pesticides with MSMA being the most likely source.

## 7. Health Risk Aspects of Arsenic Exposure

### 7.1. Public Health Perspective of Arsenic Exposure

A number of land use considerations recently have become recognized as being of interest in terms of the significance that arsenic and other substances (e.g., pesticides) may play in site-specific decisions [138]. For example, conversion of sites from agricultural use to residential, to inactive Brownfield designations, and the redevelopment of former golf courses to restricted or unrestricted residential communities, is occurring with increasing frequency. Therefore, arsenic toxicity and potential health risk are of concern in general at site-specific locations. Arsenic occurs in Florida soils as a result of natural conditions or anthropogenic processes at concentrations ranging from less than 1 mg/kg to greater than 50 mg/kg in some geographic areas [65,66,67].

The potential for arsenic toxicity is influenced by the chemical form, and also by physical/chemical properties of the specific compound in which it is present. Trivalent compounds (As^+3^; arsenic trioxide (former use in treated wood, most common arsenic form in industrial air emissions), sodium arsenite (historical herbicide and pesticide), and arsenic trichloride (chemical intermediate)) usually are judged to be more toxic than pentavalent compounds (As^+5^; arsenic pentoxide (pesticide), lead arsenate (former pesticide), calcium arsenate (former pesticide and herbicide)). The more water-soluble compounds typically exhibit greater toxicity as compared to less soluble compounds. Organic forms of arsenic (e.g., monomethylarsonic acid (MMA), dimethylarsinic acid (DMA)) are often found in the diet, and typically exert lesser toxicity than inorganic forms [139]. Soluble inorganic arsenic compounds can be absorbed through the gastrointestinal tract (>90%) and the lungs, can travel to the liver, kidney, lung, spleen, aorta, and skin, and are excreted mainly in urine [139,140,141]. While skin lesions may occur following ingestion, absorption through intact skin typically is of limited toxicological interest. Enzymatic conditions which influence arsenic metabolism and urinary arsenic ratios may be indicators of specific sensitivity in some individuals [141].

Oral doses to humans in the 50 to 100 ug/kg·day range have been reported to cause effects in some individuals following acute or intermediate duration exposures (less than one year) [139]. The acute lethal dose to humans reportedly is in the range of 10 to perhaps 125 mg/kg, compared with about 50 to 100 ug/kg·day for longer duration exposures [139]. In animals, acute oral exposures can cause gastrointestinal and neurological effects. Oral LD_50_ values range from about 10 to 300 mg/kg [139,142]. Subchronic exposure can result in immunosuppression and liver and kidney effects at fairly high doses. Chronic exposures can result in skin effects and bile duct enlargement. Some reproductive effects have been reported after prolonged exposure in animals at doses much greater than those generally achieved following incidental soil exposure.

Epidemiologic studies have shown a connection between high arsenic drinking water concentrations and increased incidences of dermal and other types of cancers [139,141,143]. A number of World Health Organization reviews have reached similar conclusions regarding consumption of groundwater exhibiting high arsenic concentrations [144,145]. Some occupational studies have shown correlations between arsenic exposure and lung cancer [139,146]. A recent detailed and wide-ranging review of arsenic toxicology evaluates many important toxicity, physiology and exposure considerations that influence the types and occurrence of potential adverse health effects [147].

### 7.2. Exposure to Arsenic in Soils and Drinking Water in Florida: Health and Regulatory Perspectives

The initial SCTLs for arsenic that were developed for FDEP were revised in 2005 to include consideration of relative bioavailability (RBA), which refers to the amount of a substance that actually can be absorbed by the body, in comparison to an administered dosage. A statewide technical review group recommended to FDEP that a relative bioavailability value of 25% was scientifically reasonable and technically justified. At the end of the process, the agency applied a somewhat more restrictive 33% RBA value to establish a residential Soil Cleanup Target Level (SCTL) for arsenic of 2.1 mg/kg. RBA is influenced by soil type, form, or site-specific or chemical factors in Florida soils, as has been reported elsewhere [13]. A recent compilation of experimental RBA values by USEPA [148] reported an average of 30% for 103 bioavailability estimates from 88 soils across multiple sites and multiple experimental animal species. The other regulatory and exposure-based inputs to the Florida residential SCTL include a 10^−6^ target risk level (1 in 1 million excess cancer risk), 30-year exposure duration, 350 days/year exposure frequency, and aggregate resident assumptions of 59 kg body weight and 120 mg/day soil ingestion.

A number of other states in the United States also have established soil evaluation criteria, some of which include consideration of RBA, alternative cancer risk levels, reported background concentrations, and other factors [138]. The cleanup levels in approximately half of the states are higher than the Florida residential SCTL with many of the cleanup levels based on natural background concentrations [149,150,151,152,153]. States with soil SCTLs higher than Florida have not found high health risk for these areas. At some sites in Florida and elsewhere, the USEPA has implemented protective, health-based soil cleanup targets of 20 mg/kg or more for residential or other unrestricted land use circumstances. Thus, while FDEP has set a conservative default residential SCTL (2.1 mg/kg) with respect to protective soil arsenic concentrations and generally low natural background concentrations, an exceedance of that level in an individual soil sample does not necessarily indicate that a hazard to human health exists.

In addition to the unrestricted use residential SCTL of 2.1 mg/kg for arsenic in soil, FDEP also has evaluated potential risk that may be posed by arsenic at school sites, and at recreational sites (e.g., playgrounds, rails-to-trails facilities) on a number of occasions. Based upon exposure assumptions that were selected to be specific to those scenarios (e.g., 180 to 210 days per year exposure), the agency has recommended that appropriate protective soil concentrations range from 5.5 mg/kg to over 20 mg/kg for school and various park environments. In addition, in 2001, the Florida Department of Health (FDOH) concluded that arsenic in soil at playgrounds at up to 10 mg/kg is not expected to result in increased cancer risk in usual circumstances [154]. The FDEP default SCTL for protection of commercial/industrial workers, assuming direct exposure 5 days per week, for 50 weeks per year, for 25 years, currently is set at 12 mg/kg total arsenic. The FDEP site cleanup framework under Chapter 62-780, Florida Administrative Code also allows for the development of site-specific risk-based alternative SCTLs, typically in conjunction with appropriate institutional controls and/or engineering controls, depending on actual site exposure circumstances.

Many studies at locations in Florida and other states and countries suggest an apparent disconnect between theoretical calculated protective levels and potential health hazards related to arsenic in soils [14,154]. One study involved the Barker Chemical Site in Inglis, Levy County, Florida, an inactive chemical facility that formerly produced phosphate fertilizer from ore that had an elevated arsenic content. Disposal of waste from that facility resulted in soil in some residential areas that was contaminated with relatively high levels of arsenic. Preliminary studies of soil in residential areas of Inglis revealed arsenic concentrations up to 3000 mg/kg. Other studies undertaken by the USEPA near the Inglis site detected arsenic concentrations in soil up to 687 mg/kg in residential areas [154]. The FDOH performed both hair and urine analyses for arsenic on 25 residents of the area, including children, as the latter were judged to have had the greatest exposure potential to surface soils. The agency reported no detectable arsenic in more than 80% of urine samples, with all of the detected values occurring within the normal reference range (<50 μg arsenic/gram creatinine). Similarly, inconsequential results were found for the analysis of arsenic in hair samples. The agency concluded that none of the tested participants had analytical results that indicated excessive exposure to environmental arsenic, and the agency recommended that no further public health activities were warranted. Thus, even at demonstrably elevated arsenic soil concentrations, noteworthy exposure and absorption could not be demonstrated.

Similar results have been reported for some sites in western states (e.g., Montana) where arsenic in soils is naturally elevated, or where local mining activity has occurred. Body burden studies in these areas generally showed limited increases, typically less than WHO evaluation guidelines for intervention, despite residential land use [14,139,155].

Conversely, the 2016 addendum to the ATSDR Toxicological Profile for Arsenic includes citations to two more recent studies conducted in Mexico and China that do suggest significant correlations between soil arsenic concentration and potential childhood health effects [141,156,157]. Authors of both of these preliminary studies acknowledged the potential limitations of their conclusions, with the Chinese study authors concluding that “The potential influences of other environmental factors cannot be ruled out, and the results in this study should only be regarded as an initial finding.”

During another Florida investigation, a number of samples of beach sand were collected in a Dade County municipality and from a variety of other beach locations along the Miami Beach barrier island system. Arsenic concentrations in the seventeen samples of local background beach sand and renourishment sand ranged from 2.0 to 11.0 mg/kg, sixteen of which (94%) exceeded the Florida arsenic default residential SCTL of 2.1 mg/kg. However, the similarity of the arsenic concentrations among the samples, and the close agreement between the measured values with those reported in the literature as background for similar coastal environments, yielded a conclusion that the measured concentrations reflected a background condition that was independent of human activities. Further, based on an evaluation of those data, FDOH concluded that there was not a significant increased health risk related to exposure to arsenic in the beach sand, even under an assumption of potential lifetime exposure [158]. These findings also are supported by studies that have been conducted by Miami-Dade County regarding arsenic in soils on coastal barrier islands [159], and regarding anthropogenic background arsenic concentrations in surface soil elsewhere in the county [64]. The barrier islands study reported the mean arsenic concentration in the top two feet of soil to be 4.5 mg/kg, and the anthropogenic background study demonstrated that the County-wide arsenic soil concentration in the top two feet ranged from 0.3 to 29.7 mg/kg with a reported mean concentration of 2.9 mg/kg and a 95% UCL concentration of 3.7 mg/kg.

As noted previously, natural arsenic levels in groundwater generally are low in Florida (median of about 1 μg/L). However, arsenic contaminated sites may include groundwater impacts that may exceed Florida guidelines for drinking water. In such cases, Florida regulations allow for consideration of non-potable uses such as irrigation, combined with institutional controls to prohibit potable or other uses. In such instances, site-specific risk considerations can be applied.

It is important to bear in mind that, because arsenic is a naturally-occurring and ubiquitous substance, humans can be exposed from a variety of sources, especially through common components of the normal diet [139,141,160,161,162]. More specific recent work [163] has concluded that seafood and some processed foods (e.g., rice, some juices) may represent forms of particular interest. WHO [144] noted that, while dietary sources of arsenic exist, in areas of the world where significant arsenic concentrations in groundwater (natural or anthropogenic) are present, the dietary sources typically are of secondary importance. ATSDR [139] states that the highest dietary levels of arsenic are found in seafood, meats and grains. Typical historical U.S. dietary levels of arsenic ranged from 0.02 mg/kg in grains and cereals to 0.14 mg/kg in meat, fish, and poultry [164]. Shellfish, crustaceans, and marine fish often can be shown to contain the highest concentrations of arsenic (average about 4 to 5 mg/kg, maximum up to greater than 100 mg/kg). However, a substantial portion of the arsenic in fish tissue is present in the virtually nontoxic trimethylated form known as arsenobetaine (≥90 of fish arsenic) [165]. There also is available evidence to suggest that arsenic at low levels is actually an essential nutrient, given that it plays an essential role in metabolic processes of man and other mammals [139,147,166,167,168], although a recommended daily amount has not been established.

When combining all of the potential sources of exposure (food, water, air, and soil), the federal Agency for Toxic Substances and Disease Registry [139] estimated that most people consume on the order of 50 μg/day of arsenic, most of which is in less toxic organic forms (e.g., methylated forms such as MMA and DMA). The database of available organic arsenic studies remains limited [141], but suggestive evidence of cancer-causing potential in mice has been presented by Tokar et al. [169,170]. It should be noted that those latter authors do acknowledge that “Further study is required to define the role of methylated species in arsenic carcinogenesis.”

In addition to the Florida-specific case studies presented in this section, national and international environmental and health organizations, as well as independent toxicologists, have evaluated the occurrence, exposure potential, and toxicology of environmental arsenic [139,141,147,165,166,171,172]. The general toxicological consensus is that, while arsenic undoubtedly has the capability in some circumstances to cause adverse health effects, including cancer, the likelihood of such effects is strongly dependent on several factors, including at least the following:

- arsenic form (inorganic, organic);
- exposure medium (soil, food, air, water);
- internal methylation and other toxicokinetic or metabolic processes (e.g., absorption, detoxification, activation);
- intake route (oral, dermal, inhalation); and,
- exposure circumstances (concentration, frequency, duration).

A combination of the foregoing factors will determine whether arsenic will exert its potential to cause toxic effects in any particular set of circumstances. It is important to recognize that the available health-based soil screening criteria explicitly are designed to represent safe concentrations, and the criterion development process incorporates a number of conservative (i.e., protective) assumptions. Thus, as noted, exceedance of a numerical screening criterion does not conclusively demonstrate that a meaningful human health hazard is present.

## 8. Discussion

### 8.1. Background Arsenic Concentrations in Drinking Water and Soils of Florida

Compared to other areas of the United States and world, the background arsenic concentrations in the natural geologic units and soils are relatively low in Florida. While the natural background of geologic units and soils are generally low in arsenic, there are vast natural areas of Florida that do have values above the arsenic SCTL of 2.1 mg/kg for residential areas. In the older geologic units that crop out in different areas of Florida, the average concentrations of arsenic range from 1.5 to 8.8 mg/kg with the Peace River Member of the Hawthorn Group having the largest average concentration at 8.8 mg/kg (Table 1). The natural background of soils in Florida has an average concentration of less than or near to 1 mg/kg (Table 2). However, the soils containing higher concentrations of organic matter tend to have higher corresponding concentrations of arsenic with the histosols being 2.06 mg/kg and the Everglades organic soils ranging from 2.82 to 3.13 mg/kg. Chen [67] found that the highest arsenic concentrations in soils was in the Everglades calcareous entisols.

Arsenical pesticides have been used in Florida since before 1900 and many soils in Florida have been treated with pesticides and fertilizers, resulting in elevated background concentrations in many areas, particularly certain citrus areas and some other specific croplands (e.g., cotton). Phosphate-based fertilizers have been used throughout Florida and all of them contain significant concentrations of arsenic which are well above natural background values in soils. Therefore, a high percentage of farmland in Florida contains enrichment in arsenic in varying degrees. This issue is insignificant where agricultural lands remain in that use, because there is no action concentration for soils in these areas, when lands are converted from agricultural use to residential use, vast areas may then fall within the regulatory criterion of 2.1 mg/kg. This issue creates a need to consider the health risks of soil exposure to arsenic in soils and whether the action standard is reasonable. Florida has a standard that ranks in the middle of the other states, but lower than most other countries which range from 5 to 150 mg/kg [138].

For the general public, there are three potential routes of exposure to arsenic in the Florida environment. Ingestion occurs via incidental contact with impacted soil, diet/foods, and in drinking water. Direct contact occurs during exposure to soils via places like play grounds, ballfields, and on beaches. Inhalation of wind-blown dust containing some arsenic can occur from sources such as dry stormwater retention ponds in the urban environment, dry wetland soils during excavation, or from fallow farms fields.

Based on the data collected for Florida on arsenic in drinking water, there is minimal risk to those using public utilities for drinking water or bottled water with a known chemical composition. However, a potential risk may exist for those using private groundwater wells that have not been tested for arsenic. While there appears to be limited concentrations of naturally-occurring arsenic in Florida groundwater, the point sources of arsenic discussed herein show that areas close to or downgradient from golf courses or another source of groundwater contamination with arsenic could be at risk. Private wells in areas of known groundwater impacts or that have naturally high background concentrations of arsenic (e.g., the Peace River Member of the Hawthorn Group), could also have elevated dissolved arsenic concentrations, possibly above the MCL of 10 μg/L.

### 8.2. Inconsistencies of Public Policy with Regard to Health Risk Assessments and Regulatory Actions Involving Arsenic in Florida

In Florida, the legislative mandate of a one-in-one million target risk level “under actual circumstances of exposure” (Section 376.30701(2), Florida Statutes), combined with widely variable but generally low natural background soil levels, has resulted in regulatory actions that often do not reflect the limited health concerns of soil arsenic at levels up to 20 mg/kg or more (see Section 7.2). The USEPA has evaluated a number of arsenic contaminated sites in Florida and has recommended cleanups at soil concentrations of 5.5 to 20 mg/kg, well above the Florida residential SCTL of 2.1 mg/kg. The FDEP has evaluated arsenic concentrations on school playgrounds containing 5.5 mg/kg of arsenic without invoking remedial actions. At Inglis, Florida, residential soil arsenic concentrations were found by the USEPA to be up to 687 mg/kg. A FDOH investigation of 25 people living in that area showed no significant health impacts of the arsenic. On Miami-Dade County beaches, arsenic was found in the sand at concentrations ranging from 2.0 to 11.0 mg/kg with 94% of the samples exceeding the SCTL of 2.1 mg/kg. No remedial action was required in this case. In these cases, either site-specific risk evaluations or other studies were performed to establish alternative cleanup levels or to determine that concentrations were naturally-occurring. In some cases where anthropogenic sources of arsenic occurred at sites in low to moderate concentrations, cleanups have occurred which involved socio-political decisions that may not be health-risk based (e.g., City of Fort Myers site).

There are cases where naturally-occurring soil concentrations of arsenic occur adjacent to or within residential areas that are above the 2.1 mg/kg SCTL. Isolated or jurisdictional wetland soils commonly have the highest arsenic concentrations in the natural environment of Florida, some of which exceed the 2.1 mg/kg SCTL for residential areas. It is also likely that some conservation lands, a land use that does not have an established SCTL for arsenic, lie directly adjacent to residential lands which typically would require compliance with the default SCTL. The default SCTL invariably would be lower than the arsenic concentrations in the conservation lands, and an expensive, time consuming background study may be required.

## 9. Conclusions

Arsenic occurrence is ubiquitous in the natural rocks and soils of Florida at concentrations that can be significant. Within the geologic units underlying Florida, from Eocene to Miocene in age, the natural arsenic concentrations in these predominantly carbonate rocks ranges from 1.8 to 8.8 mg/kg. Within these rocks at the small scale, grains of the mineral pyrite occur that can have arsenic concentrations well over 1000 mg/kg. For comparison purposes, the FDEP regulatory guidelines for arsenic remedial action is 2.1 mg/kg for residential properties and 12 mg/kg for commercial and industrial properties.

Many areas of Florida contain soils that exceed the SCTL residential standard of 2.1 mg/kg due to a combination of natural and anthropogenic added arsenic. Natural soils in Florida have an average natural concentration of arsenic of less than 1 mg/kg, but the organic soil types (histosols) have a higher concentration at an average of 2.06 mg/kg. Organic soils within the Everglades have an even higher arsenic concentrations ranging between 2.82 and 3.13 mg/kg based on the average of numerous samples and considering seasonal variations. Certain calcareous entisols in the Everglades have even higher arsenic concentrations. The soils of Florida have also been enriched in arsenic due to the use of arsenic in pesticides, fertilizers, soils amendments, and various other types of contaminants, such as the creation, use and disposal of Chromated Copper Arsenate (CCA)-Treated Wood, disposal of water treatment plant sludges, disposal of chicken litter, and other sources.

While detailed toxicological information has been compiled to set the United States and the World Health Organization drinking water standards for arsenic, the human health risk posed by arsenic occurrence in rocks and soils has been debated in various venues. Default cleanup criteria based on “safe” or background concentrations vary greatly by state and by country. However, all regulatory agencies allow for the determination of cleanup arsenic concentrations using site specific data that incorporate the many variables that affect risks associated with exposure to arsenic. In Florida, the SCTL is 2.1 mg/kg for residential area soils. However, site-specific risk assessments typically result in significantly higher site-specific cleanup concentrations.

A review of the arsenic data on soils in Florida shows that it is very sparse in many areas. Data are also sparse or nonexistent for many shallow geologic units that weather into surface soils and may be used to supply drinking water via municipal and private shallow wells. Potential arsenic occurrence in untreated potable water withdrawn from private wells, especially in areas where shallow sediments and geologic units are known to contain arsenic, is worthy of further evaluation. As more information regarding the occurrences of arsenic in the natural environment and in areas impacted by humans is obtained, the bioavailabilities of the various forms of arsenic, and the behaviors of people in various residential environments will become better understood. This will necessitate that the default cleanup concentrations for arsenic and other contaminants will continue to be refined. In Florida, the SCTLs have not been evaluated since 2005. Therefore, as with other contaminants such as carcinogenic polynuclear aromatic hydrocarbons, perhaps the SCTLs for arsenic should be revisited by FDEP in the near future. Reevaluation of the SCTLs for arsenic and other legacy contaminants is in the public interest as residential development encroaches into agricultural and other potentially impacted lands.

## Figures and Tables

**Table 1:** Naturally-occurring arsenic concentrations in Florida geologic formations (complied from Miami-Dade County [64]; Lazareva and Pichler [58]; Price and Pichler [29]; Pichler et al. [60]; Unpublished data from Florida Geological Survey).

Geologic Unit	Sample Type	No. Samples	Mean Value (mg/kg)	Range in Values (mg/kg)	Standard Deviation
Miami Limestone	Bulk rock	22	<0.2		
Hawthorn Group	Bulk rock total	362	5.6	0.1–69.0	7.1
	Interval	285	5.0	0.1–40.8	5.8
	Special interest	77	8.3	0.4–69.0	10.5
	Pyrite	126	1272	<1–8260	1379
	Bulk rock total ^1^	142	3	<1–33	4
Undifferentiated Arcadia Formation	Bulk rock total	205	5.7	0.1–36.0	6.2
Tampa Member	Bulk rock total	75	3.0	1.2–15.2	3.7
	Pyrite	31 (in 1 sample)		10–2180	
Nocatee Member	Bulk rock total	27	6.5	0.5–69.0	13.1
Peace River Fm.	Bulk rock total	55	8.8	0.4–40.8	8.6
Suwannee Limestone	Bulk rock total	306	3.5	0.1–54.1	7.4
	Interval	235	1.7		2.8
	Targeted	71	9.5		12.5
	Pyrite	25	2300	100–11,200	2700
	Bulk rock total ^1^	61	2	<1–6	1
Ocala Limestone	Bulk rock total	70	1.5	<0.1–14.7	2.9
	Bulk rock total ^1^	58	2	<1–23	3
Avon Park Formation	Bulk rock total	373	2.2	<0.1–30.8	4.2
	Interval		1.0		
	Targeted		3.2		
	Pyrite	228	945	100–5820	1026
	Bulk rock total ^1^	41	3	<1–10	3

^1^ The bulk rock samples were homogenized and analyzed for arsenic by the Florida Geological Survey.

**Table 2:** Concentrations of arsenic is Florida soils with pH, clay content, and organic carbon concentrations (from Chen et al. [63]; Ma et al. [65]).

Soil Type	As (mg/kg)	pH	Clay Content (%)	Organic Carbon (g/kg)	Bulk Density (mg/m^3^)
Histosols	2.06 ± 2.41	4.62 ± 1.30	NA	341 ± 15.6	0.28 ± 1.64
Mollisols	0.74 ± 3.29	6.07 ± 1.18	11.8 ± 2.61	43.2 ± 25.1	1.03 ± 1.42
Inceptisols	1.12 ± 6.22	5.13 ± 1.23	6.19 ± 3.14	22.1 ± 32.1	1.17 ± 1.50
Ultisols	0.57 ± 3.00	5.25 ± 1.19	2.11 ± 2.86	14.9 ± 25.8	1.30 ± 1.25
Entisols	0.41 ± 4.24	5.18 ± 1.21	1.77 ± 3.36	9.3 ± 20.3	1.40 ± 1.13
Alfisols	0.36 ± 3.41	5.11 ± 1.14	2.92 ± 2.41	10.1 ± 21.2	1.41 ± 1.14
Spodosols	0.18 ± 3.23	4.46 ± 1.16	1.15 ± 2.37	15.5 ± 22.4	1.28 ± 1.18
Correlation coefficients (r-value)	-	0.14	0.33	0.58	-

This study baseline range 0.01–50.6 mg/kg for 445 samples; Ma et al. [65] reported baseline of 1.1 mg/kg.

**Table 3:** Arsenic concentrations in the Everglades area [67].

Season	Environment	Mean (mg/kg)	Range (mg/kg)
Dry Season	Soil	2.82 ± 1.97	0.142–8.41
	Floc	4.41 ± 2.45	0.84–13.7
	Periphyton	1.26 ± 1.00	0.22–4.06
Wet Season	Soil	3.13 ± 2.77	0.074–14.9
	Floc	3.39 ± 1.91	0.49–8.74
	Periphyton	2.12 ± 1.79	0.38–7.17

**Table 4:** Arsenical pesticide chemical registered for use in Florida [12].

Active Ingredient	Use
Monosodium acid methanearsonate	Herbicide
Calcium acid methanearsonate	Herbicide
Cacodylic acid	Herbicide
Cacodylic acid, sodium salt	Herbicide
Arsenic trioxide	Ant killer
Disodium methanearsonate	Herbicide
Sodium arsonate	Herbicide
Arsenic pentoxide	Wood preservative
Arsenic acid	Wood preservative, biocide

192 products are registered in Florida using these ingredients.

**Table 5:** Compilation of arsenic concentration data in lime sludge in Florida [116].

Sample Location	Total Arsenic Concentration, mg/kg	Leachable Arsenic (μg/L)
Arcadia Water Department	0.29	<2.5
Bonita Springs Water System	0.20	<2.5
Charlotte County Utilities	2.13	<2.5
City of Cocoa	0.31	<2.5
City of Englewood	0.40	<2.5
Flagler Beach WTP	0.43	<2.5
Murphree WTP (Gainesville)	0.80	<2.5
City of Lakeland	0.82	<2.5
City of North Lauderdale	0.95	<2.5
Lauderdale Lakes BCOES 1A	0.20	<2.5
Manatee County Public Works	4.93	<2.5
Florida Water Services—Marco Island	0.69	<2.5
North Brevard County/Mims	2.44	<2.5
OAK	2.04	<2.5
City of Ocala WTF	0.80	<2.5
Pompano Beach BCOES 2A	0.47	<2.5
City of Pahokee	3.69	<2.5
Fort Pierce Utilities	0.37	<2.5
St. Johns County (CR-214)	0.18	<2.5
St. Johns County (CR-214)	0.73	2.84
Average	1.15	
Standard Deviation	1.28	
Minimum	0.18	<2.5
Maximum	4.93	2.84
